# A Thyrotoxicosis Surprise: Jod-Basedow Phenomenon Following IV Contrast Administration

**DOI:** 10.7759/cureus.24742

**Published:** 2022-05-04

**Authors:** Akriti Pokhrel, Moe M Tun, Serajus S Miah, Jilmil S Raina, Tooraj Zahedi

**Affiliations:** 1 Internal Medicine, Brookdale University Hospital Medical Center, Brooklyn, USA; 2 Medicine, Bangladesh Medical College, Dhaka, BGD; 3 Endocrinology, Brookdale University Hospital Medical Center, Brooklyn, USA

**Keywords:** contrast, iodine, thyroid, wolff chaikoff effect, jod-basedow effect

## Abstract

Jod-Basedow phenomenon (JBP) is a rare thyrotoxic condition due to increased exogenous iodine exposure, also known as iodine-induced hyperthyroidism (IIH). Historically JBP was typically seen in iodine-deficient patients when exposed to increased amounts of iodine. However, in today's era, the most common cause of JBP is exposure to iodinated contrast media commonly used in various radiological examinations and interventional procedures, resulting in massive iodine exposure. Patients with normal thyroid function usually experience no ill effects. There has been increasing use of iodinated contrast in imaging and procedures over recent decades. Deposition of iodine in the thyroid in a person with normal functioning thyroid glands would usually be autoregulated and inhibited by the Wolff Chaikoff effect. However, a small albeit a significant portion of patients, particularly those with pre-existing thyroid conditions, can escape this auto-regulatory effect and be subject to life-threatening conditions, such as arrhythmias, heart failure, pulmonary arterial hypertension, cerebrovascular and pulmonary embolism, and cardiomyopathy. We present a case of a 59-year-old female with pre-existing goiter who presented with altered mentation and seizures, requiring endotracheal intubation for airway protection. She underwent a CT angiogram of the head and neck for a suspected stroke, receiving iodinated IV contrast in the process. Thyroid function tests on admission showed a thyroid-stimulating hormone (TSH) of 0.974 mIU/L (reference range 0.465-4.650 mIU/L) and free T4 of 0.46 ng/dL (reference range 0.75-2.19 ng/dL). The ensuing ICU course was complicated by thyrotoxicosis eight days after contrast administration with a surge of free T4 from 0.46 ng/dL on admission to 4.07 ng/dL and a TSH suppression to <0.015 mIU/L. She subsequently required three sessions of emergent plasmapheresis to remove excess free T4 before undergoing partial thyroidectomy and cardiac catheterization. Iodine-induced hyperthyroidism solidifies the need for awareness of a potential JBP following contrast administration, especially in an aging population and undiagnosed thyroid conditions, and timely diagnosis and intervention can greatly influence outcomes.

## Introduction

Jod-Basedow phenomenon (JBP) is a rare thyrotoxic condition due to increased exogenous iodine exposure, also known as iodine-induced hyperthyroidism (IIH). Historically JBP was typically seen in iodine-deficient patients when exposed to increased amounts of iodine. However, one of the most common causes of JBP is exposure to iodinated contrast media commonly used in various radiological examinations and interventional procedures, resulting in massive iodine exposure. Patients with normal thyroid function usually experience no ill effects. However, in contrast, those with pre-existing thyroid disease may experience thyrotoxicosis, which, if unrecognized, could lead to serious, potentially life-threatening consequences, such as atrial only arrhythmias, heart failure, pulmonary arterial hypertension, cerebrovascular and pulmonary embolism, and cardiomyopathy. With this, we present an interesting case of a 59-year-old female with a history of goiter (uninvestigated before this presentation), who developed JBP after receiving intravenous iodine contrast for computed tomography angiogram (CTA) head and neck and required plasmapheresis to manage hyperthyroidism. CTA was done on our patient to rule out cerebrovascular accident (CVA) and carotid artery stenosis, as she presented with altered mental status (AMS). This case raises awareness of the risks of JBP due to iodinated contrast agents routinely used for our aging patient population.

## Case presentation

**Figure 1:**
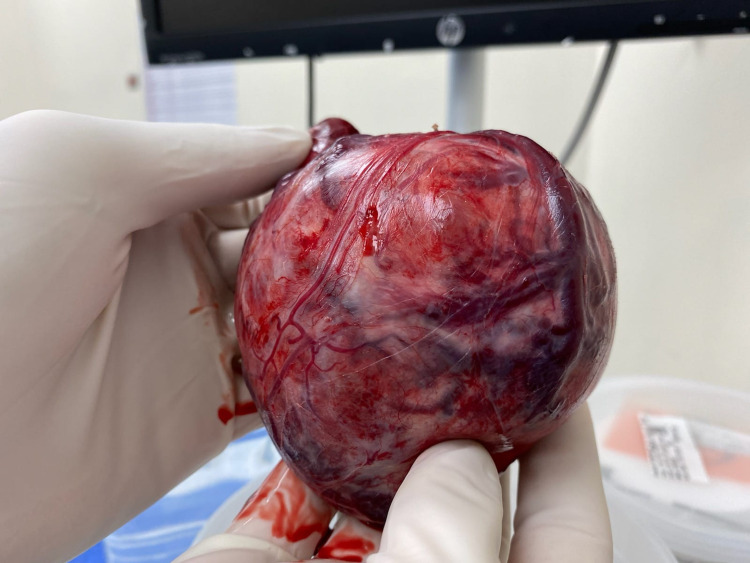
This image shows the thyroid gland of the patient post isthmusectomy, a sample was sent for histopathological monitoring.

A 59-year-old female, with a past medical history of heroin abuse (as per the patient she discontinued heroin use a couple of years ago before presentation) and goiter, initially presented with AMS and new-onset seizure, requiring endotracheal intubation for airway protection, with the help of video laryngoscopy. Initial thyroid function test showed thyroid-stimulating hormone (TSH) 0.974 mIU/L (normal), free T4 0.46 ng/dL (low). CTA head and neck with iodine contrast was done to rule out stroke as a reason for AMS and seizure. Neurology was consulted for AMS and seizure and the seizure was believed to be apparently a new-onset seizure with no recurrence and under control with levetiracetam. The result was negative for stenosis, aneurysm, or dissection of intracranial vessels but showed massive thyroid enlargement including a 9-cm dominant right thyroid mass with left side tracheal deviation. The patient developed sinus tachycardia after the CT angiogram with iodine contrast and TSH was <0.014 mIU/L and free T4 of 4.07 ng/dL. The patient was diagnosed with a thyrotoxic crisis due to JBP. Emergent plasmapheresis was planned to reduce free T4. The patient was also treated with Dexamethasone 2 mg IV every six hours while continuing with sessions of plasmapheresis. Sinus tachycardia resolved. After two sessions of plasmapheresis, TSH was <0.015 mIU/L, free T4 decreased to 1.11 ng/dL. The patient underwent diagnostic cardiac catheterization for non-ST elevation myocardial infarction (NSTEMI). After the procedure, one more session of plasmapheresis was done to prevent thyrotoxic crisis again. TSH was <0.015 mIU/L, but free T4 was elevated to 2.21 ng/dL after cardiac catheterization. She could not be extubated throughout the hospital course due to external compression of the trachea by goiter. The patient underwent right thyroid lobectomy and isthmusectomy (Figure 1) after a few days in which, the patient was successfully extubated. TSH was still <0.015 mIU/L but free T4 decreased to 0.85 ng/dL (normal). Biopsy results showed encapsulated papillary thyroid carcinoma with a predominant follicular growth pattern.

## Discussion

Over the years, the use of iodine-containing contrast media has been on the rise in imaging studies. A standard dose of IV contrast containing 13,500 μg of free iodide and 15-60 g of bound iodide could be converted to free iodide.

JBP usually self-resolves within weeks to months of the inciting event. Management includes the use of Beta-blockers to prevent the development of significant hyperthyroid symptoms. However, in severe cases of hyperthyroidism, methimazole can be used. For our patient, Isovue-300 was used as a contrast agent. This is Lopamidol 61%, with each ml containing 612 mg of iopamidol with 1 mg tromethamine and 0.39 mg edetate calcium sodium. This contains 0.043 mg sodium and 300 mg organically bound iodine per ml, which is several times higher than the daily recommended allowance. Some nodules in the thyroid are independent of TSH regulation. Excessive amounts of iodine can induce increased hormone production from these follicles. Even 300 μg of iodide can induce JBP, an amount which is less than in iodine-containing contrast media with risks increasing in the elderly and those with underlying multinodular goiter or Graves' disease.

The mechanism of autoregulation, when exposed to iodine excess, means increased iodine leads to increased transport. The resulting expansion of the iodine pool causes decreased hormone synthesis and autoregulatory inhibition of iodine transport, known as the Wolff Chaikoff effect [1]. Defective or absent autoregulatory mechanisms lead to pathologic consequences of iodine excess [2]. Failure to escape from the Wolff Chaikoff effect leads to sustained inhibition of hormonal synthesis resulting in an increase in TSH and enlargement of the thyroid gland, leading to goiter and hypothyroidism. These two conditions are self-limiting and subside when iodine levels return to normal. JBP occurs when there is excess iodine and absent autoregulation. In this condition, excess iodine leads to a sustained increase in hormone synthesis and, eventually, thyrotoxicosis. It is characterized by decreased radioactive iodine uptake due to an expanded iodine pool and inhibition of TSH stimulation [3].

Unfortunately, our patient was required to undergo surgery for persistent hyperthyroidism and her free T4 level was trending down gradually thereafter.

Iodine-induced thyrotoxicosis or JBP has been reported as iodine is used to treat endemic goiter throughout the world. These days, iodinated radiocontrast agents and antiarrhythmic drugs such as amiodarone are the most common causes of the JBP, especially in elderly patients with underlying thyroid disease or multinodular goiter. Iodine is considered essential for thyroid hormone synthesis and requires approximately 52 µg. The recommended daily intake is 150 µg with a high threshold of 1100 µg. The average daily intake in the USA is 150-200 µg, which makes it an iodine-replete country [4].

The mechanism of the Jod-Basedow phenomenon is related to chronic stimulation of the thyroid gland by TSH causing mutations in follicular cells. This results in the production of large quantities of thyroid hormones when iodide becomes available in sufficient amounts as is in cases of iodinated contrast administration [5].

Our patient had normal thyroid-stimulating immunoglobulin (89%, normal value <140%), and no clinical evidence of Graves' disease at the time of presentation, hence ruling out Graves' disease as a cause. The patient had no recent symptoms of viral illness leading up to her hospitalization. On exam, thyroid glands were non-tender, rendering viral thyroiditis unlikely. Patients on amiodarone, lithium, interferon-α, and interleukin-2 can present with painless thyroiditis, however, our patient was not on any of these medications.

Cocaine use, although not a known cause of hyperthyroidism, has been shown to be associated with it and can potentially result in overt clinical hyperthyroidism in patients with underlying subclinical hyperthyroidism. Although our patient's urine drug screen was positive for cocaine use, she did not have subclinical hyperthyroidism as evident from her thyroid panel performed two months prior to admission.

There are reported cases in the literature of patients with metastatic follicular cancer developing thyrotoxicosis, however, thyroid malignant tumors are rarely associated with hyperfunctioning thyroids. The development of thyrotoxicosis in our patient, who was previously euthyroid prior to contrast administration, points to the diagnosis of JBP in a patient with papillary thyroid cancer, a very rare yet eye-opening presentation.

## Conclusions

With the increasing use of contrast imaging in the modern era in an ever-aging population and many potentially undiagnosed goiter cases, the above article serves as a very important reminder to keep an eye out for the potential development of JBP, detection, and early intervention which can prevent arrhythmias, congestive heart failure, embolic stroke, and even death. The use of contrast for both elective and emergent imaging will only increase. This begs the question as to whether thyroid evaluation before and after imaging for at-risk patients can become a new norm going forward.
